# Determinants of temperature sensitivity of soil respiration with the decline of a foundation species

**DOI:** 10.1371/journal.pone.0223566

**Published:** 2019-10-17

**Authors:** Danielle D. Ignace

**Affiliations:** Department of Biological Sciences, Smith College, Northampton, MA, United States of America; Tennessee State University, UNITED STATES

## Abstract

The eastern hemlock (*Tsuga canadensis*) is an important foundation species that is currently declining throughout eastern U.S. forests due to the exotic pests hemlock woolly adelgid (*Adelges tsugae*) and elongate hemlock scale (*Fiorinia externa*). Hemlock is often replaced by deciduous tree species, such as black birch (*Betula lenta*), and has been shown to have large consequences for carbon dynamics due to a substantial loss of soil organic layer carbon storage in hemlock forests when replaced by birch and higher decomposition found in black birch stands. Soil carbon is one of the most important components of the global carbon cycle and has high potential to feedback to climate change when large portions of stored carbon are lost to the atmosphere. There is a general consensus that soil respiration increases with temperature, but there has yet to be a consensus on how temperature sensitivity of soil respiration is affected by various biotic and abiotic factors, such as soil moisture and substrate quality. In this study, the effects of soil temperature and soil moisture on soil respiration (*R*_s_), the temperature sensitivity of soil respiration (*Q*_10_), and soil basal respiration (*R*_10_) were investigated for hemlock, young birch, and mature birch forest types annually for three years. The *R*_s_ values of the three forest types were primarily driven by soil temperature rather than by soil moisture across all years. Soil respiration data collected from hemlock, young birch, and mature birch stands were used to determine annual *Q*_10_ and *R*_10_ values. The *Q*_10_ and *R*_10_ values were not significantly different between forest stands, but they were significantly different over the three years. Determinants of *Q*_10_ and *R*_10_ differed between forest type, with soil moisture primarily influencing *Q*_10_ in hemlock and mature birch stands and soil temperature primarily influencing *R*_10_ in mature birch stands. The results suggest a complex interaction of soil moisture and soil temperature, and potentially substrate quality and quantity, as determinants of temperature sensitivities in eastern U.S. forests that have transitioned from hemlock-dominated to black birch-dominated forests.

## Introduction

The world is witnessing an unprecedented acceleration in warming trends, which may have devastating implications for ecological process [1]. One of the greatest challenges facing scientists is predicting how ecosystems will respond to a warming climate. Despite numerous studies that have devoted efforts to addressing this problem there is currently no consensus on projecting future shifts in soil respiration rates with a warming climate. Soil respiration (*R*_s_, flux of carbon dioxide (CO_2_) from the soil to the atmosphere) represents one of the largest fluxes of the global carbon cycle [2] and it has been estimated that soil respiration (plant root and microbial) contributes ~90 Pg C yr^-1^ [3]. However, a comparison of models demonstrated a range of global *R*_s_ from 83 to 108 Pg yr ^-1^ [4]. Soil CO_2_ flux to the atmosphere is important, because these estimates can outweigh fossil fuel emissions by an order of magnitude [5–7]. Some studies show the global soil-to-atmosphere (total soil respiration) carbon dioxide flux is increasing [3,5]. In particular, the ratio of heterotrophic respiration in surface soil layers to total soil respiration has increased globally over recent decades [8]. Contrary to single-site studies, global synthesis data has not found evidence of acclimation of soil respiration to warming [9]. Due to the complex nature of the interaction of other factors influencing soil respiration, including soil moisture, carbon (C) substrate levels, quality of carbon substrate, and nutrient availability [10–12], deciphering how rising temperatures will affect soil respiration rates remains a difficult task.

Exploring temperature sensitivity of soil respiration in eastern US forests is important, as this region is losing a foundation tree species, the eastern hemlock (*Tsuga canadensis*). Hemlock forests provide a great model system to explore temperature response of soil respiration, as these ecosystems are facing widespread destruction due to the exotic insect pests hemlock woolly adelgid (*Adelges tsugae*, HWA) and elongate hemlock scale (*Fiorinia externa*, EHS). It is important to understand the implications of hemlock loss as these forests have vitally important roles in the eastern US, such as their strong effects on ecosystem characteristics, unique associations with other organisms, and their function as a sink for atmospheric CO_2_ [13–19]. Changes in the leaf litter characteristics, soil organic layer mass, carbon:nitrogen (C:N) content, and soil respiration rates have been monitored for several years at the MacLeish Field station, located in Whately, MA, USA [20]. This work took advantage of an “accidental experiment” initiated by patch-level timber harvesting ~30 years ago, which allowed us to investigate changes in carbon (C) storage as hemlocks are replaced by black birch. This work showed a significant reduction in soil organic layer mass and C:N values as hemlock forests transition to mature birch forests [20]. Most importantly, this led to a 6.8× decline in soil organic layer C storage and a net release of ~4.5 tons C per hectare, as supported by significantly higher soil respiration in the mature birch stands [20]. This is a novel finding as there has been little evidence to date of these forests becoming significant sources for atmospheric CO_2_ [21,22]. Additionally, previous work has documented substantial changes in decomposition and nitrogen cycling as we see the loss of hemlocks [21–25].

In the current study, I sought to address how the temperature sensitivity of soil respiration (*Q*_10_) and basal rate of soil respiration (*R*_10_) changes as eastern hemlock forests transition to mature black birch forests. Three main questions were addressed in this study: (1) how do *Q*_10_ and *R*_10_ values vary as forests transition from hemlock to mature birch stands, (2) how do *Q*_10_ and *R*_10_ values display inter-annual variation, and (3) how does soil moisture and soil temperature influence *Q*_10_ and *R*_10_ values? To address this, I estimated *Q*_10_ and *R*_10_ values across the 2015–2017 growing seasons. Determining temperature sensitivities of soil respiration has gained considerable attention in recent years and studies have suggested that *Q*_10_ is highly variable [26,27]. It has been suggested that the quality and degradability of soil organic carbon is an important regulator of *Q*_10_ and that recalcitrant soil organic carbon is more sensitive to warming than fresh and labile organic matter [11]. Additionally, there is no clear understanding how different quality substrates will respond to increasing temperatures [11]. Hemlock trees have low foliar and litter N, which results in slow rates of decomposition, soils with low extractable N pools and low rates of potential net mineralization and nitrification [25,28]. Slow rates of nutrient cycling creates a deep and acidic organic layer with high C:N [17]. Taken together, these stands have high potential to be carbon sinks in the northeastern US. Since we observed an incremental decrease (hemlock > young birch > mature birch) in soil organic layer mass and its C:N in a previous study [20], I predict *Q*_10_ and *R*_10_ values to follow the same incremental decrease. Since the dense canopy of hemlock stands creates a cooler environment with lower light levels in the understory compared to deciduous stands [16], then I also expect the young and mature birch stands to have soil moisture as the main factor influencing temperature sensitivities.

## Materials and methods

### Study site description

Data collection for this study was completed in a forested area at Smith College’s Ada and Archibald MacLeish Field Station, located in Whately, MA (N 42° 27.32’, W 72° 40.96) in central New England. A full site description of the MacLeish Field Station is in Ignace et al. (2018), although a few important details are mentioned here. The field station is characterized as having 105-ha of largely secondary growth Northern Hardwoods-Hemlock-White Pine forest [18] and HWA and EHS have been observed on hemlocks at the MacLeish field station since 2009–2010. Nine plots (10 × 15 m) were established, with three plots in each of the forest types. Patches of young black birch trees were established ~25–30 years ago due to selective logging of hemlock patches in 1988 [18]. Based on tree core sample data, the two mature forest types (hemlock, black birch) are ~80–100 years in age and those in the young birch plots are 25–30 years old (J. Bellemare personal communication). The mature birch plots are ~100 m east of the hemlock and young birch plots. The hemlock forest type is dominated by hemlock (58.9% of basal area), and includes some black birch (33.6%) and other deciduous tree species (7.6%) [20]. The young birch plots are dominated by black birch (83.4% basal area) and the mature birch plots are dominated by black birch (78.7%) [20]. Barring drought conditions, soil moisture (VWC, %) is typically the highest in young birch plots, with peak values of 35% in July of 2015, 25% in June of 2016, and 21% in May of 2017 [20].

### Soil measurements

Soil respiration is the total soil CO_2_ efflux, which encompasses heterotrophic and autotrophic respiration. Soil respiration data was collected every ~3–4 weeks throughout the growing season (~May to November) during three consecutive years (2015–2017). In 2015, the sampling started May 27 and ended October 23. In 2016, the sampling started May 23 and ended November 4. In 2017, the sampling started May 24 and ended November 27. A closed-path infra-red gas analyzer system (LI-6400; LI-COR Inc., Lincoln, NE USA) was used to measure the rate of soil respiration. Each forest type consisted of three 10-cm diameter polyvinyl chloride (PVC) soil collars. On a given sampling date, three measurements were made on each soil collar between 10am and 2pm. If necessary, above-ground material on top of the soil collar was removed. Soil temperature was measured concurrently with soil respiration using a temperature probe attachment on the LI-6400. Adjacent to each soil collar, volumetric water content measurements were made with a soil moisture probe (HydroSense, Campbell Scientific Inc., Logan, UT USA) at a depth of 12 cm.

### Data analysis and statistics

The mean soil efflux (*R*_s_) and soil temperature from the three collars in each plot was used to determine the temperature response of soil respiration. Equation 1 represents the *apparent Q*_10_, not actual, because it is the temperature response when used on data at the seasonal to annual time scale [10].

*Q*_10_ is the multiplicative change in rate per 10°C change in temperature.

The response of soil respiration rate measured in the field, *R*_*s*_, to soil temperature, *T*_*s*_, was assessed using the linearized *Q*_10_ function [29], which is the natural-log transformation:

$$ln\left(R_{s}\right)=A+BT_{s}$$

The A and B regression coefficients were then used to estimate the apparent *Q*_10_ of soil respiration:

$$Q_{10}=exp\left(10B\right)$$

Soil basal respiration was calculated by:

$$R_{10}=Q_{10}\times exp\left(A\right)$$

Long-term *Q*_10_ and *R*_10_ values were estimated using the annual data for each forest type. Variations in *Q*_10_ and *R*_10_ among forest type were analyzed using one-way ANOVA. A linear regression was used to determine the relationship between these estimates and soil temperature and volumetric moisture. A nested ANOVA was used to analyze differences in mean soil respiration, soil temperature, and volumetric water content over time, forest type, and their interaction. All analyses and graphing were conducted in R Version 3.3.3 [30].

## Results

### Soil temperature

**Fig 1:**
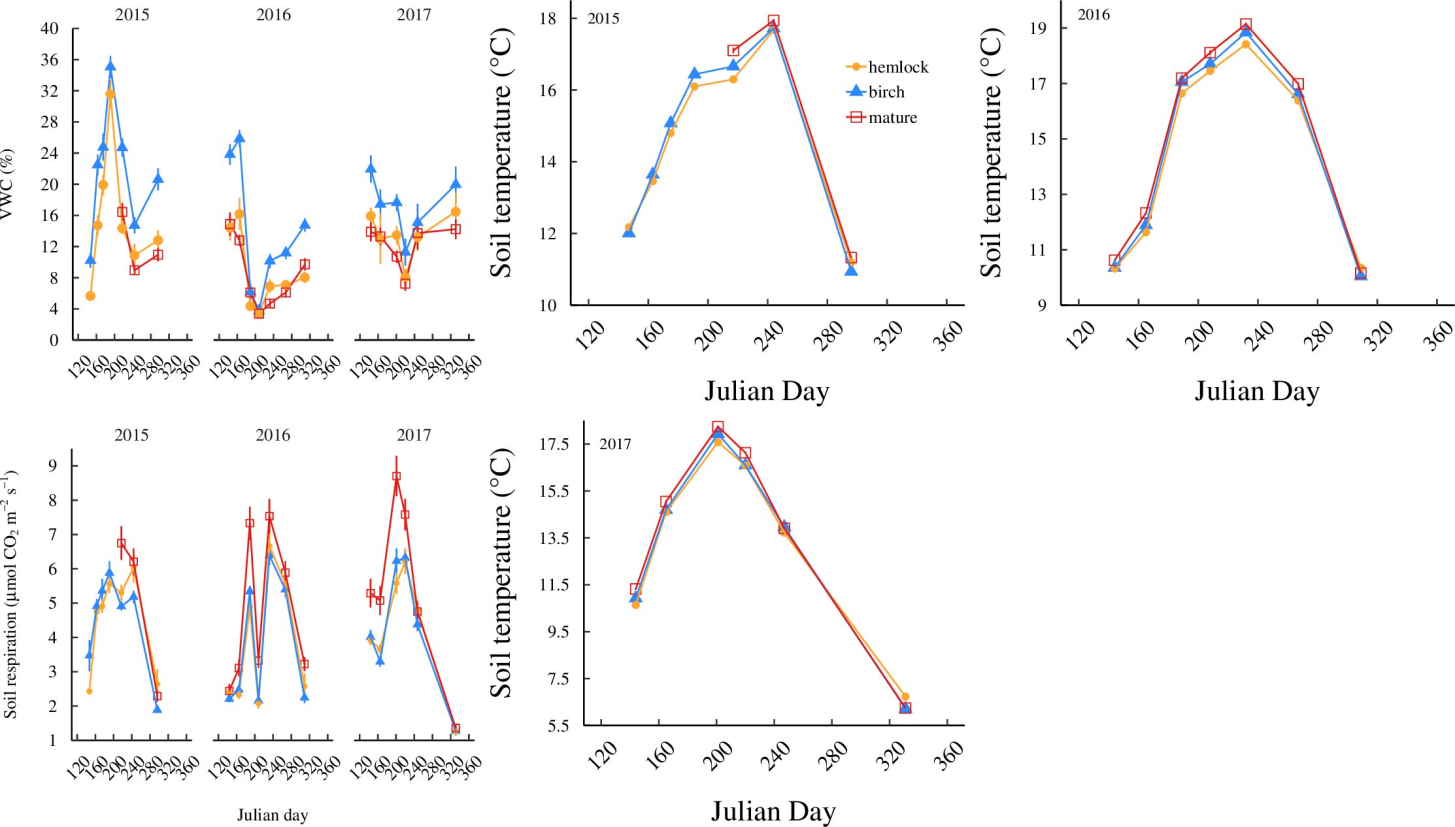
Trends of soil temperature, soil moisture, and soil respiration within a growing season of each year. Mean volumetric moisture (VWC %) (A), *R*_s_ (B) and soil temperature (C) for each forest type over Julian day in 2015, 2016, and 2017. Error bars are ± 1 standard error. Hemlock has yellow circles. Young birch has blue triangles. Mature birch has red squares.

A repeated measures ANOVA was used to analyze soil temperature within each year (Fig 1A). Soil temperature was significantly different by date (*F*_1,416_ = 13.970, *P* = 0.0002), and by a forest and date interaction during the 2015 growing season (*F*_2,416_ = 60.850, *P* < 0.0001). During the 2016 growing season, soil temperature was significantly different by date (*F*_1,510_ = 25.942, *P* < 0.0001). During the 2017 growing season, soil temperature was significantly different by date (*F*_1,384_ = 20.234, *P* < 0.0001) and by a forest and date interaction (*F*_2,384_ = 5.866, *P* = 0.003). Generally, soil temperature was highest in the mature birch, followed by young birch, followed by hemlock plots. These differences are most apparent during the summer growing season and converge during the late spring and early fall. All forest types displayed the lowest temperature seen in late spring and fall and the highest temperature seen in mid summer.

### Volumetric water content

Volumetric soil moisture (Fig 1B) was significantly different by forest type (*P* < 0.0001) during the 2015 growing season [20]. The 2016 growing season was marked with drought conditions with significant differences by forest type (*P* < 0.0001) and date (*P* < 0.0001) [20]. The 2017 growing season showed no significant differences [20]. During non-drought conditions, young birch plots consistently had the highest soil moisture at each sampling date.

### Soil respiration

Soil respiration (*R*_s_, Fig 1C) was significantly different by a forest type and date interaction (P < 0.0001) during 2015, by date only (P < 0.0001) in 2016, and by date (*P* < 0.0001) and the forest and date interaction (*P* < 0.0001) in 2017 [20]. Overall, *R*_s_ for all forest types were the lowest pre- and post-growing season. Hemlock and young birch had similar *R*_s_ throughout the most active periods of the growing season. Mature birch always had the highest *R*_s_, reaching differences of as much as 60% greater than hemlock rates during the middle of the growing season in all years [20]. More specifically, post-hoc analysis revealed significant differences on many samples dates. Mature birch had the highest (*P* < 0.05) soil respiration in August of 2015, all sampling dates (except May) of 2016, and all sampling dates (except September and November) of 2017.

### The relationship between soil respiration and the soil environment

**Fig 2:**
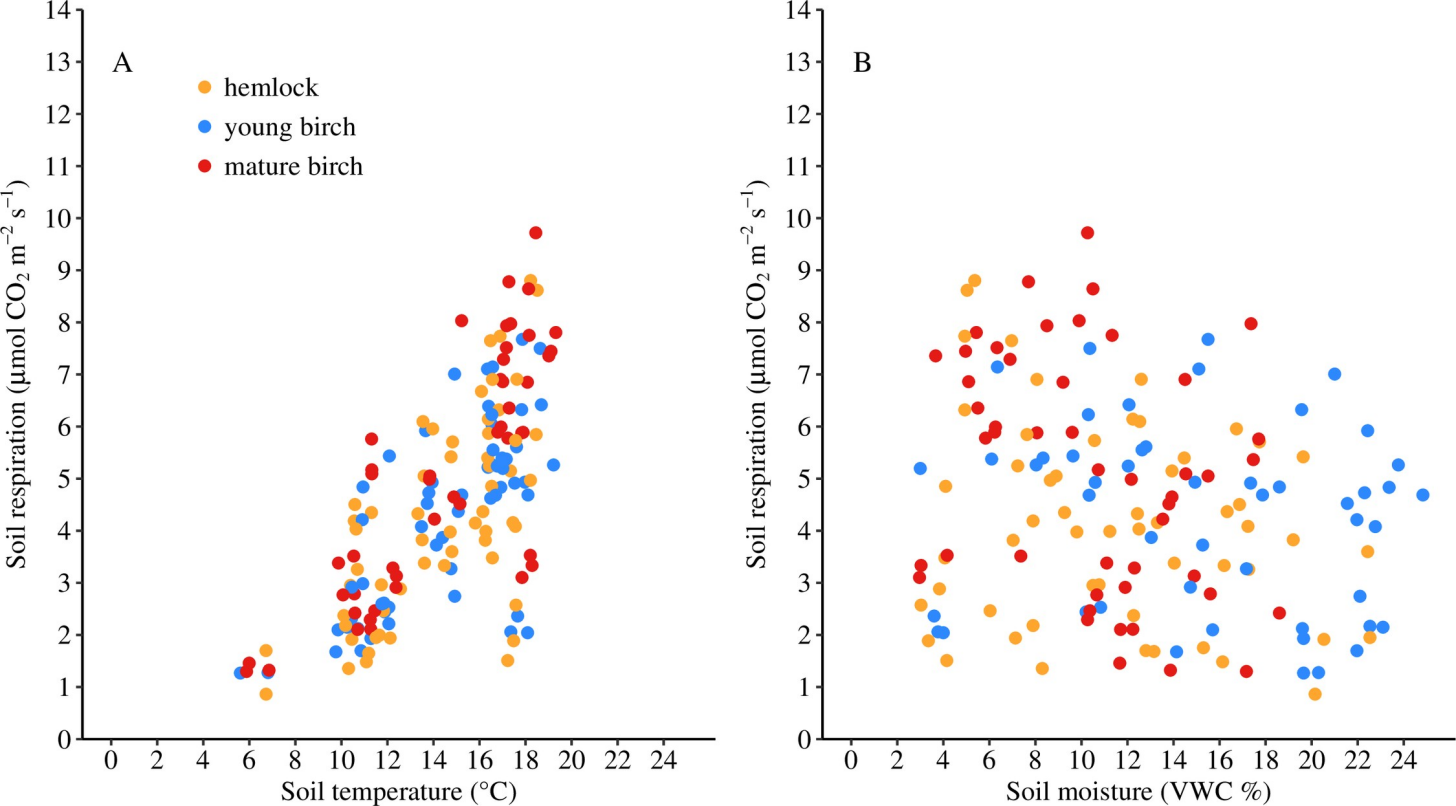
The relationship between soil respiration and the soil environment. The relationship between *R*_s_ and (A) soil temperature (°C) and (B) soil moisture (volumetric water content, VWC %) for each forest type from 2015–2017. All points are mean values per soil collar. The yellow, blue, and red points represent hemlock, young birch, and mature birch stands, respectively.

Measurements of *R*_s_, soil temperature, and soil moisture measurements at each collar across all sample dates from 2015 to 2017 were used to determine the relationship between soil respiration and the soil environmental factors. Overall, *R*_s_ increased exponentially with soil temperature (*R*^2^ = 0.44, *P* < 0.0001, Fig 2A). There was no significant relationship between *R*_s_ and soil moisture (Fig 2B).

### Annual variation in temperature sensitivity and basal rate of soil respiration

**Fig 3:**
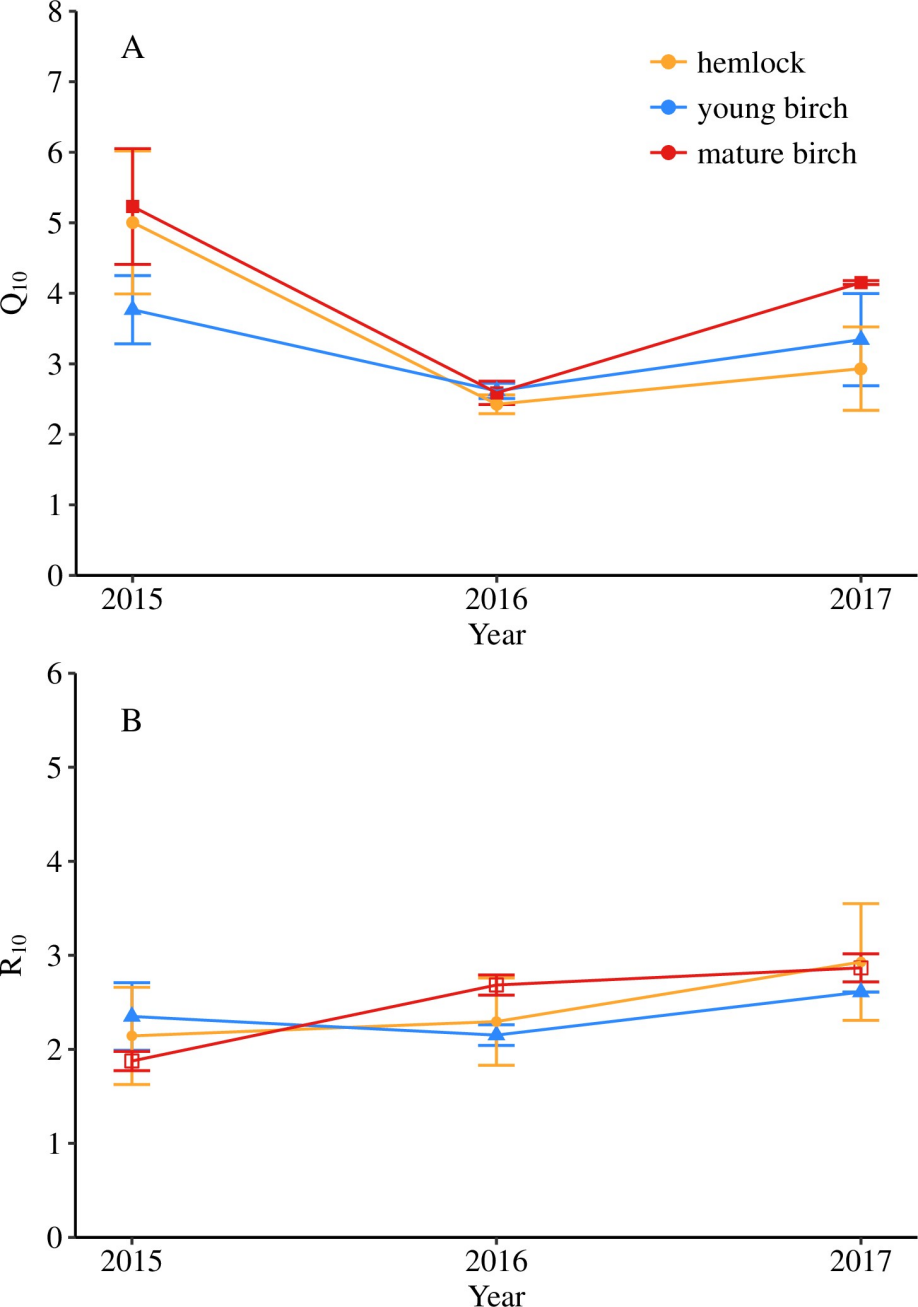
Values of *Q*_10_ and *R*_10_ for each year. Inter-annual variation of *Q*_10_ and *R*_10_ for each forest type over Julian day within 2015, 2016, and 2017. Error bars are ± 1 standard error. Hemlock has yellow circles. Young birch has blue triangles. Mature birch has red squares.

Using the dataset collected during 2015 to 2017 from the three forest types, temperature sensitivity (*Q*_10_) was obtained. Temperature sensitivity of soil respiration (*Q*_10_) was close, albeit not significantly different by year (*F*_1,17_ = 4.320, *P* = 0.055, Fig 3A), and not significantly different by forest type. Within in each forest type, *Q*_10_ was highest in 2015 and lowest in 2016 (Fig 3A). Hemlock stands had *Q*_10_ values range from 2.43 to 5.00, young birch stands had values range from 2.62 to 3.77, and mature birch stands had values range from 2.59 to 5.23 (Table 1).

**Table 1:** Values of *Q*_10_ and *R*_10_. Mean *Q*_10_ and *R*_10_ (mean ± SE) for each forest type in the 2015, 2016, and 2017 growing season (n = 3).

**Stand**	*Q*_**10**_	*R*_**10**_
***2015 Season***		
**Hemlock**	5.00 ± 1.01	2.14 ± 0.52
**Young birch**	3.77 ± 0.48	2.35 ± 0.36
**Mature birch**	5.23 ± 0.82	1.88 ± 0.10
***2016 Season***		
**Hemlock**	2.43 ± 0.13	2.30 ± 0.46
**Young birch**	2.62 ± 0.11	2.15 ± 0.11
**Mature birch**	2.59 ± 0.17	2.68 ± 0.11
***2017 Season***		
**Hemlock**	2.93 ± 0.59	2.93 ± 0.62
**Young birch**	3.34 ± 0.65	2.61 ± 0.00
**Mature birch**	4.15 ± 0.03	2.87 ± 0.15

The *R*_10_ values were significantly different by year (*F*_1,17_ = 18.801, *P* = 0.0004), but not by forest type. The *R*_10_ values showed a trend of increasing year after year (Fig 3B). Generally, the range of *R*_10_ values were much more narrow than the ranges found for *Q*_10_. Hemlock stands had *R*_10_ values range from 2.14 to 2.93, young birch stands had values range from 2.15 to 2.61, and mature birch stands had values range from 1.88 to 2.87 (Table 1).

### The influence of the soil environment on temperature sensitivity of soil respiration

**Fig 4:**
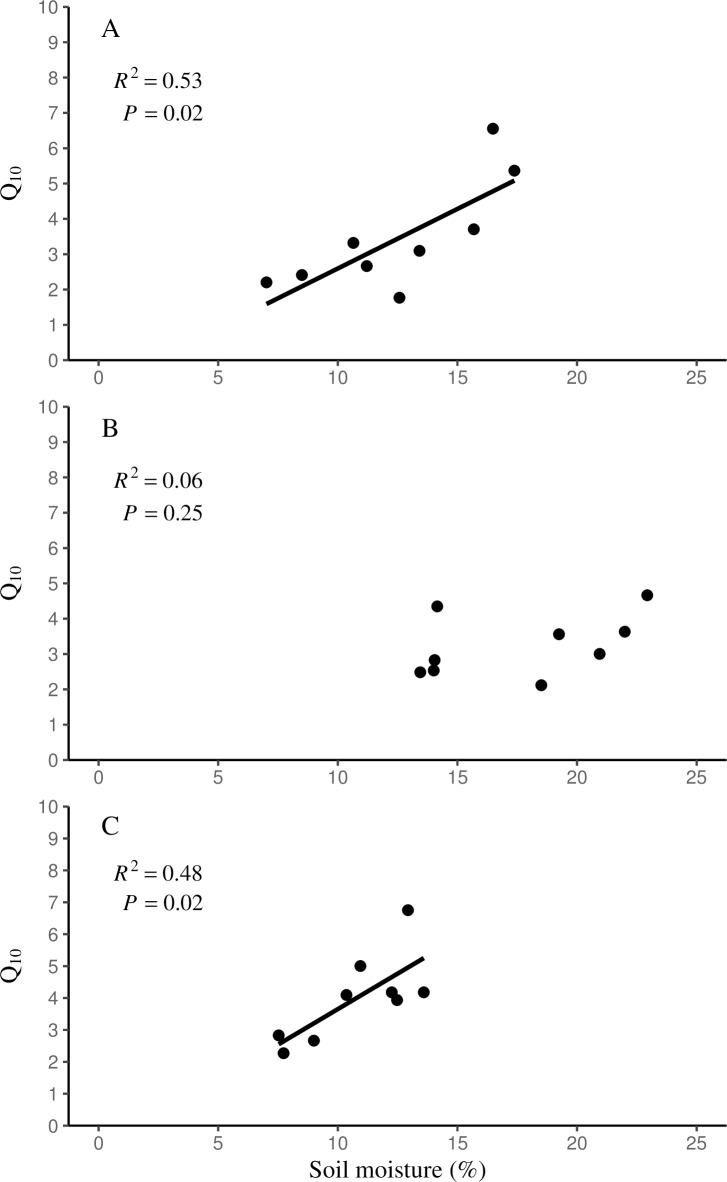
The relationship between *Q*_10_ and soil moisture. The relationship between *Q*_10_ and soil moisture (volumetric water content; VWC %) for hemlock (A), young birch (B), and mature birch stands (C) across all years.

A linear regression was used to fit the relationship between *Q*_10_ and soil moisture across all years (Fig 4). There was a significant relationship found between *Q*_10_ values and soil moisture in the hemlock stands (*R*^2^ = 0.53, *P* = 0.02, Fig 4A) and the mature birch stands (*R*^2^ = 0.48, *P* = 0.02, Fig 4C), but no relationship was found in the young birch stands (Fig 4B). There was no relationship of *Q*_10_ and soil temperature.

### The influence of the soil environment on basal rate of soil respiration

**Fig 5:**
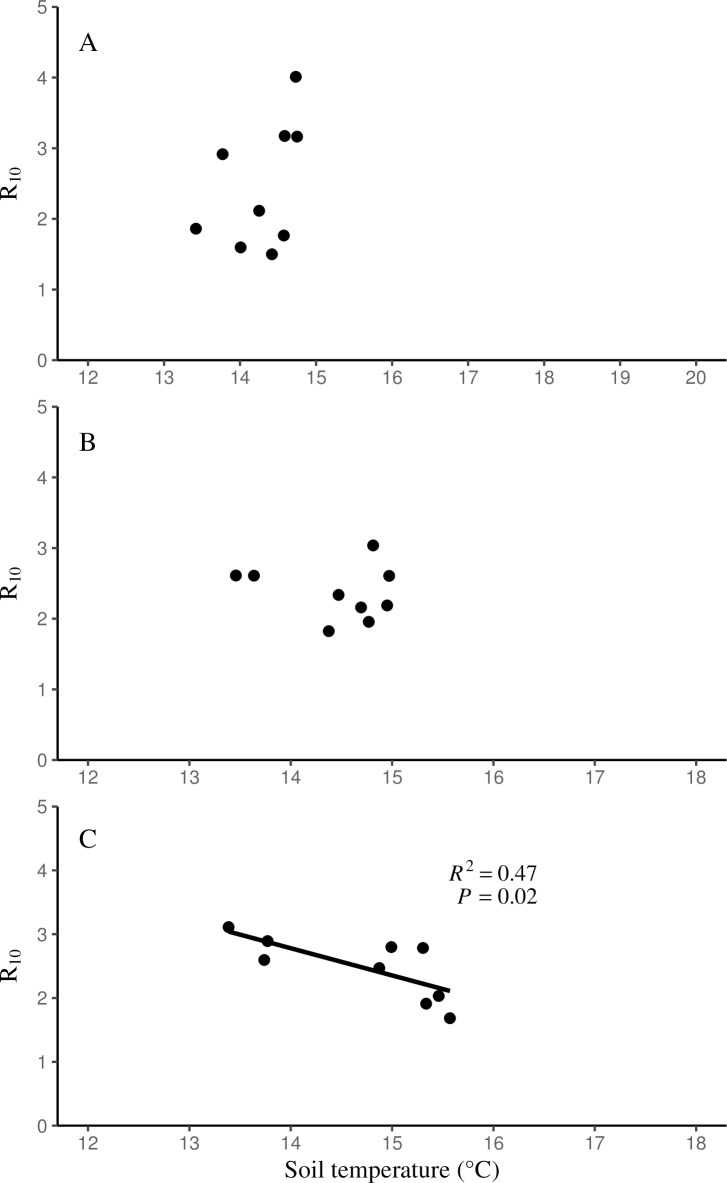
The relationship between *R*_10_ and soil temperature. The relationship between *R*_10_ and soil temperature (°C) for hemlock (A), young birch (B), and mature birch stands (C) across all years.

There were no significant relationships found between *R*_10_ values and soil moisture. Mature birch stands were the only forest type that showed a significant relationship of *R*_10_ values and soil temperature across all years (*R*^2^ = 0.47, *P* = 0.02, Fig 5C).

## Discussion

Three years of data collection were used to investigate the temperature sensitivity (*Q*_10_) and basal soil respiration rate (*R*_10_) of soil microbial activity within hemlock, young birch, and mature birch stands in western Massachusetts. Previous work at this field site demonstrated that as hemlock forests transition to mature birch forests, there was a significant increase in *R*_s_, a massive reduction in the soil organic layer, and a decrease in the soil C:N of the soil organic layer [20]. Based on these findings, I expected the three different forest types to exhibit drastically different temperature sensitivities of soil respiration with mature birch exhibiting the highest level of temperature sensitivity. In the present study, *R*_s_ increased exponentially with increasing temperature, with 53% of the variation explained by this relationship, while *R*_s_ was not correlated with soil moisture. Results also showed no significant differences in *Q*_10_ and *R*_10_ across forest types, but they differed significantly over the three years. More importantly, the determinants of *R*_10_ and *Q*_10_ varied between forest types, with the *Q*_10_ of mature hemlock and birch stands influenced by soil moisture and the *R*_10_ of the mature birch stands influenced by soil temperature. The following discussion considers how the results could be due to differences in annual environmental conditions, soil conditions, C:N of the soil organic later, and an interactive effect of soil conditions and fungal and bacterial communities.

### The soil respiration relationship with temperature and soil moisture

The significant positive relationship between *R*_s_ and temperature is consistent with previous studies [5,6,31], as temperature can be the most important factor regulating global soil respiration [4]. Even though certain biomes have soil moisture and soil carbon emerging as dominant predictors of soil respiration [4], soil moisture was not found to be a good predictor of *R*_s_ in the current study. There may be several reasons for this outcome. The young birch plots consistently demonstrated higher soil moisture values relative to the hemlock and mature birch plots throughout the entire season of every year. Meanwhile, the hemlock and mature birch plots demonstrate very similar soil moisture across all sampling dates for every year. This difference is likely due to lower overall biomass and soil moisture needs of young birch plots (~25–30 years old) compared to the mature hemlock and birch plots aged ~80–100 years. During drought conditions, all forest types converge to the same soil moisture content. In contrast to the soil moisture data, *R*_s_ was significantly higher in the mature birch plots, and thus must be primarily driven by factors other than soil moisture. Values of *R*_s_ increased exponentially with increasing temperature in the current study, but this is less than the 83% of variation explained in a similar system [22]. Even though *R*_s_ was drastically different between hemlock and mature birch stands in previous work at the MacLeish field site [20], there is considerable variation in *R*_s_ within each plot that could be responsible for this. Additionally, the 2016 growing season was an abnormally dry year (459.5 mm of precipitation from April to October), while 2015 and 2017 had levels of precipitation that were more typical for this region [20]. When the 2016 growing season data was excluded from the analysis, there was 67% of the variation in *R*_s_ that was explained by soil temperature (*P* < 0.0001). However, there may be other factors, such as substrate quantity and quality, which may account for less variation explained compared to other studies.

### The variation in Q_10_ and R_10_ and their determinants

There was substantial variation in annual values of *Q*_10_ in the present study, although it was not statistically significant. It is important to discuss how the variation in the current study compares to other studies and delve into potential explanations for this outcome. Significant differences in temperature sensitivities were expected since previous work demonstrated an incremental decrease in organic layer C:N (26.46 in hemlock > 23.54 in young birch > 20.21 in mature birch) as hemlock forests transition to mature birch forest [20]. Other research in central Massachusetts and south-central Connecticut showed no significant difference in organic layer %C between hemlock and mature birch forests plots [32], thus it was not as surprising to see that they also found no significant difference in *Q*_10_ among stands [22]. The average annual range of *Q*_10_ values (between 2 and 5 for each soil collar) in the present study were similar to what has been observed in Finzi et al. (2014) who found *Q*_10_ varied from 1 to 9. On average, the *Q*_10_ of hemlock stands in this study was higher than values of found in other studies, and the *Q*_10_ of black birch stands were lower [22,33]. While Drake et al. (2013) found substantial variation in *Q*_10_ across the different fluxes and forest types, including hemlock stands and other stands dominated by other plants (1-red oak and red maple, 2- white ash) in central Massachusetts [34]. Results from these other studies and results in the present study show the potential for large differences in canopy and soil conditions to elicit large differences in *Q*_10_.

It is possible that variation in soil moisture can directly affect *Q*_10_ values in this system. Variations in *Q*_10_ have been shown to be substantial even at similar soil moisture levels from a broad range of ecosystems in North America [35]. In the present study, there was little variation in the *Q*_10_ values among forest types during the 2016 growing season. This was an unusually dry season highlighted with periods of drought, thus limiting water supplies likely influenced such low *Q*_10_ values. A significant relationship was found between *Q*_10_ and soil volumetric water content (VWC), but significance was not found in every forest type. The hemlock and mature birch forest types had similar VWC, while the young birch stands always maintained the highest VWC throughout the 2015 to 2017 growing seasons [20]. This was likely due to the younger birch stands having less biomass compared to the hemlock and mature birch stands, leaving more water available in the soil. The young birch stands did not show any relationship between *Q*_10_ values and soil moisture, while the hemlock and mature birch stands both showed soil moisture as a significant determinant of *Q*_10_. Since the hemlock and mature birch stands have lower soil moisture than young birch stands, then soil moisture may be more susceptible to increases in temperature and limit plant activity. Trends in soil temperature during each growing season were very similar across forest type, but were significantly different by a forest and time interaction, except during the 2016 growing season. There was no significant relationship found between *Q*_10_ values and soil temperature, but the possibility exists that soil temperature may indirectly affect *Q*_10_ values by affecting soil moisture availability in mature stands of hemlock and birch.

Other factors like substrate availability might be a better determinant for *Q*_10_ values. Although not directly manipulated in this study, previous work at this site lends support to this possibility. The young birch stands are 25–30 yr old and have significantly less soil organic material than the hemlock stands, but significantly more than the mature birch stands [20]. The mature birch stands at this site are similar in age to the hemlock stands (~80–100 yr old), but have drastically different soil organic layer mass and C:N [20]. It has been highly debated whether the temperature sensitivity of soil organic carbon decomposition varies with substrate quality (labile vs. recalcitrant) [11,36–42]. Evidence from a broad range of ecosystems demonstrates that more biogeochemically recalcitrant organic matter results in a greater temperature sensitivity of soil respiration [35]. Since substrate quality can exhibit a strong influence on temperature sensitivities of soil respiration [11,43], I expected similar factors to influence the temperature sensitivities in this study. The hemlock stands have more persistent soil organic carbon, due to a thick organic layer (4.1 cm; [44]). Though not formerly tested, hemlock forests are thought to have more recalcitrant organic matter, as they may be comprised of needles containing high concentrations of lignin and polyphenolic compounds, as well as humic compounds [20]. Given the environmental conditions of hemlock forests, there has been greater accumulation of organic material relative to black birch stands [20,44]. The mature birch stands have a 6.8× decline in soil organic layer carbon storage due to their higher decomposition of soil organic material with lower C:N [20]. Other studies indicate that increasing substrate availability can lead to an increase in *Q*_10_ and that an addition of a readily available C substrate significantly increases *Q*_10_ values for all soil types [10,43]. It has been hypothesized that low-quality substrate or recalcitrant soil should have a higher temperature sensitivity compared to soils with more labile substrate [42,45]. Variations of temperature and soil water content may also indirectly affect respiration via their effects on substrate availability [10]. Increases in soil temperature and moisture could accelerate decomposition of the organic layer mass, which could increase the activity of the plant roots through increased growth when nutrients become more available. Despite other work suggesting biogeochemically recalcitrant organic matter being more sensitive to temperature, there has yet to be a consensus on the generality of this relationship.

Overall, there may be more complex biotic and abiotic interactions at play that can affect temperature sensitivity of soil respiration. Communities of bacteria and fungi can be altered, as the shift from hemlock to black birch stands are related to environmental factors in addition to host specificity [46–50]. Fungal and bacterial activities and communities are also likely playing a role in driving differential organic layer accumulation in this system since these communities are influenced by soil acidity [21]. Results from the MacLeish field station has shown that soil pH was lower in hemlock stands than black birch stands [20], which mimics the conditions found in these forest types in Connecticut [51–53]. Acidic soils have been shown to have a high ratio of percent fungal to bacterial respiration [54], and soil bacterial communities can be linked to forest litter characteristics and soil pH [55]. Fungal vs. bacterial communities should be greatly affected by the transition from hemlock stands to black birch stands, since previous research found nitrogen availability and C:N of the soil as a strong influencing factor [44]. Other work on this study site showed that macrofungal communities of the forest floor did not demontrate strong differences in morphospecies richness in hemlock stands compared to black birch stands, but had more rare fungi taxa [44]. Even though bacterial community richness has not been quantified at this study site, the data show the abundance of bacterial colony forming units in the soil organic horizon follow a seasonal pattern that differs between hemlock and black birch stands [44]. Specifically, the timing of peak abundance of bacteria showed that abundance found within hemlock stands peaks in the summer and abundance found within black birch stands peaks in the fall [44]. Taken together, the bacteria could primarily be tracking the input of new inputs of leaf litter of black birch stands in the fall that is more enriched in nitrogen than hemlock stands, as opposed to directly matching peaks in soil respiration rates.

In order to better standardize soil respiration, *R*_10_ was used to understand its relationship with different soil conditions. Although *R*_10_ did not significantly differ by forest type, *R*_10_ was significantly different over time. The range of *R*_10_ values were much more narrow than the ranges found for *Q*_10_, with hemlock stands ranging from 2.14 to 2.93, young birch stands ranging from 2.15 to 2.61, and mature birch stands ranging from 1.88 to 2.87. The range of *R*_10_ values are in sync with other hemlock studies, in which *Q*_10_ estimates were more broadly distributed than the *R*_10_ estimates [22,33]. The mature birch stand was the only forest type that demonstrated a significant, and negative, correlation of *R*_10_ and soil temperature. Additionally, the mature birch stands have the widest range in soil temperatures and the highest maximum soil temperature. This is likely due to dense canopies, low light levels, and cooler temperatures that are characteristic of hemlock stands [16]. It is important to highlight the negative relationship between soil temperature and *R*_10_, as mature birch stands will potentially be the most negatively impacted forest type with rising air temperatures in the coming decades. The broad range of soil temperature currently observed may lead to more extreme fluctuations in soil temperature due to the deciduous nature of mature birch stands. Given that *R*_10_ was not correlated with soil moisture, the *R*_10_ values are mostly a result of soil temperature and potentially soil temperature in combination with substrate availability or quality.

## Conclusions

The decline of eastern hemlock in eastern U.S. has large ramifications for ecosystem processes as these forests are often replaced by the deciduous black birch trees. Hemlock forests transitioning to mature birch forests at the MacLeish Field Station served as a model system to investigate the implications for temperature sensitivity and basal rate of soil respiration and their determinants. Field measurements of soil respiration were mostly controlled by soil temperature, and not soil moisture. Trends of *R*_10_ and *Q*_10_ were significantly different over time for, but were not different among forest types. Nonetheless, the determinants of *R*_10_ and *Q*_10_ varied between forest types and were likely influenced by their soil characteristics. Previous work at this field site has shown that as hemlock stands transition to mature birch stands, C storage decreases, soil respiration rates increase, C:N of the soil organic material decreases, macrofungal communities may become homogenized, and the timing of peak bacterial abundance may shift to the fall [20,44]. Even though temperature sensitivity of soil respiration did not concurrently shift, it is important to note that mature birch plots trended towards having the highest *Q*_10_ values relative to the young birch and hemlock plots, which runs counter to previous suggestions that soils with slower turnover have more sensitivity to a warming climate [11,35,39]. The *Q*_10_ values were influenced by soil moisture in mature stands of hemlock and black birch, but not the young black birch stands. While mature birch was the only forest type that showed a significant influence of soil temperature on *R*_10_ values. Taken together, continued monitoring of years that incorporate drought and high precipitation seasons will be important for understanding the response of soil moisture to continued temperature warming. Like previous work, it may take decades to observe alterations in the temperature sensitivity of soil respiration during the transition from hemlock to mature birch stands. Given predicted warming predictions [1], this work highlights how the temperature sensitivity of soil respiration will be vitally important to determine the potential feedbacks between decomposition, loss of soil organic layer C storage, and warming temperatures [11,56]. This will be particularly important as scientists aim to predict changes in carbon fluxes when ecosystems will be faced with a changing climate.

## Supporting information

S1 File Supplementary data. Mean soil temperature (°C), mean volumetric water content (%), *R*_10_ values, and *Q*_10_ values for every plot within every forest type.
(CSV)
